# A comparative evaluation of alectinib for ALK-positive non-small-cell lung cancer: A systematic review

**DOI:** 10.1097/MD.0000000000041506

**Published:** 2025-02-14

**Authors:** Monica Kharat, Harshitha Nookala, Jabez David John, Tirath Patel, Pragnesh Kalyandrug, Sonya Emmanuel, Anaya Noor, Dhruv Jignesh Kumar Halani, Abhishek Goyal, Nikhilesh Anand, Bharathi Gadad, Richard M. Millis

**Affiliations:** a Department of Medicine, Icahn School of Medicine at Mount Sinai, Queens Hospital Center, New York, NY; b Department of Medicine, Mamata Academy of Medical Sciences, Telangana, Hyderabad, India; c Department of Medicine, Malla Reddy Institute of Medical Sciences, Telangana, Hyderabad, India; d Department of Medicine, Trinity Medical Sciences University School of Medicine, Ribishi, Saint Vincent and Grenadines; e Department of Medicine, California Unit of Behavioral Science and Psychology, Fairfield, CA; f Department of Medicine, Dow Medical College, Dow University of Health Sciences, Karachi, Pakistan; g Department of Medicine, Windsor University School of Medicine, Basseterre, St Kitts; h Department of Neurology, JFK University Medical Center, Edison, NJ; i Department of Medical Education, University of Texas Rio Grande Valley, Edinburg, TX; j Department of Pathophysiology, American University of Antigua, Antigua and Barbuda, Saint John.

**Keywords:** alectinib, ALK-positive, chemotherapy, non-small-cell lung cancer

## Abstract

**Background:**

Lung cancer, a significant global health challenge, notably the non-small-cell lung cancer (NSCLC) subtype, is a topic of utmost importance. The continuous advancements in NSCLC treatment, especially in the context of anaplastic lymphoma kinase (ALK)-positive NSCLC, are of great interest. A thorough review of alectinib’s comparative efficacy and safety with other treatment modalities is a crucial step, and the role of clinicians and surgeons is integral in optimizing patient care. This review can also inform neoadjuvant therapies and enhance surgical education, facilitating more informed decision-making processes between surgeons and patients.

**Methods:**

This comprehensive systematic review results from rigorous screening. Following a rigorous screening process of the PubMed, PubMed Central, and Medline databases by quality assessment and application of inclusion/exclusion criteria filters, 9 relevant articles were identified that directly addressed the research question and provided a holistic understanding of it. The analysis included a total of 1403 patients from 9 different studies. Alectinib was given to 836 patients, while 567 patients received other chemotherapeutic drugs. The primary objective of this study was to evaluate and compare the efficacy of alectinib with other treatment modalities.

**Results:**

The analysis revealed that alectinib is promising for ALK-positive NSCLC cases, with significantly better efficacy and a positive impact on limiting central nervous system metastases. Alectinib’s favorable safety profile, with medically manageable adverse events, provides reassurance about its safety compared with other treatment modalities.

**Conclusions:**

Alectinib has emerged as a viable, significantly superior treatment option for patients with ALK-positive NSCLC. The superior efficacy and manageable safety profile are significant; it remains a novel therapy with much potential, such as neoadjuvant therapy, which will make significant strides in patient care of ALK-positive NSCLC. Therefore, it is crucial for healthcare professionals, including surgeons, to be well-versed in alectinib and its potential. This knowledge will empower them and instill confidence in their ability to provide the best care for their patients.

## 1. Introduction

**Figure 1.**
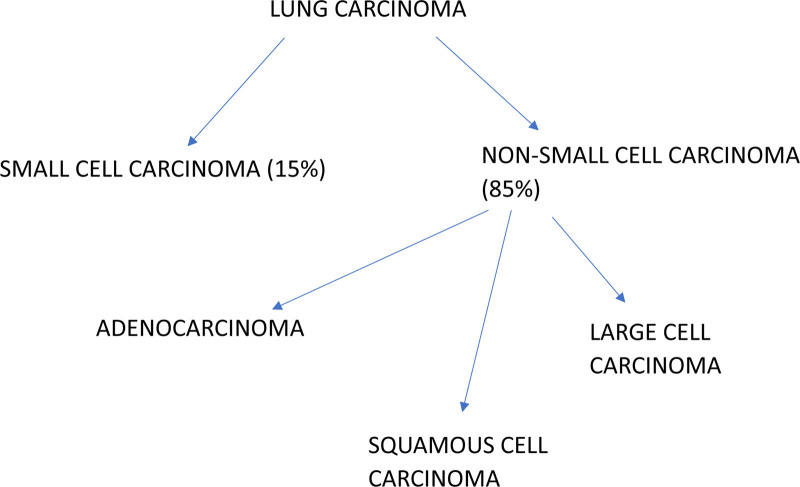
The classification of lung carcinoma, lung cancer is broadly divided into 2 main categories: SCLC and NSCLC. SCLC accounts for ≈15% of all lung cancers, while NSCLC represents the majority (85%). NSCLC is further subcategorized into 3 main types: adenocarcinoma, squamous cell carcinoma, and large cell carcinoma. NSCLC = non-small-cell lung carcinoma, SCLC = small-cell lung carcinoma.

Lung cancer is the second most common type of cancer occurring worldwide, affecting 2 million people every year, and over 1 million people die due to lung cancer each year.^[1-3]^ About two-thirds of diagnoses of NSCLCs occur at an advanced or metastatic diseased stage; this makes the treatment planning approach more complex.^[4]^ The subtypes of non-small-cell lung cancer (NSCLC) are diverse (Fig. 1).

The treatment of NSCLC is not a one-size-fits-all approach. Depending on the stage of the disease, a range of options is available, offering reassurance to patients. The treatment approach for stages I, II, and III involves personalized interventions such as surgery, chemotherapy, immunotherapy, radiotherapy, or a combination of modalities. In the case of stage 4 disease, systemic therapy is the treatment of choice due to its comprehensive care approach.^[5]^

The gold standard treatment for the early stages of NSCLC is surgical resection, which consists of a lobectomy, pneumonectomy, and sublobar resection. In recent times, segmentectomy is an alternative as it is a recently established method associated with similar oncologic and overall outcomes refractory to lobectomy. The main aim of surgical management is to achieve complete resection with negative margins and appropriate clearance of lymph node basins.^[6]^ Surgery has improved the patient’s health status more efficiently than other treatment modalities.^[7]^

For patients with high surgical risks or the ones not willing to a surgery, stereotactic body radiation therapy^[8]^ is considered the standard of care during early stages compared with other radiation therapies like external beam radiation, intensity modulated radiation therapy, brachytherapy for its improved LF rates, overall survival (OS), extremely high rates of local control, and low rate of treatment-related side effects.^[9]^ However, stereotactic body radiation therapy is the standard form of treatment only in specific populations, as it can result in the concurrent development of fibrotic lung parenchyma in response.^[8]^

In standard clinical practice, clinicians combine various treatment modalities with chemotherapy. Depending on the cancer stage and other factors, chemo can be recommended along with surgery, as it can shrink the tumor, making it easier to remove (neoadjuvant chemotherapy) and also as an adjuvant to kill the residual cancer cells along with radiotherapy. In cases of metastases of the tumor to areas like bones, liver, and adrenal gland, chemotherapy is the treatment modality of choice. One major limitation is that chemotherapy is not considered a standard mode of treatment for elderly individuals and those with poor overall health. Furthermore, it is associated with a median OS of ≈6 months, as indicated by a study.^[10]^

NSCLC is a broad category that encompasses various subtypes of lung cancer, each distinguished by different genetic mutations and characteristics. One notable subtype is anaplastic lymphoma kinase (ALK)-positive NSCLC, which is defined by the presence of an abnormal ALK gene rearrangement. This genetic alteration drives uncontrolled cell growth by activating the ALK protein, leading to tumor development. In contrast, other NSCLC subtypes may involve different genetic mutations, such as epidermal growth factor receptor mutations, Kristen RAt sarcoma virus mutations, or ROS1 fusions, each triggering distinct signaling pathways related to cancer progression.

Patient demographics also vary; ALK-positive NSCLC tends to affect younger individuals, often nonsmokers or light smokers, while other NSCLC types can occur in a broader patient population, including heavy smokers.

ALK-positive NSCLC responds effectively to targeted therapies known as ALK inhibitors which are tailored to inhibit the activity of the mutated ALK protein. Alectinib, one of these inhibitors, has gained prominence due to its long-term efficacy, high selectivity, and central nervous system (CNS) active ALK-inhibiting activity.^[11]^ It is important to note that alectinib is not the only drug in this category; other options include brigatinib, crizotinib, ceritinib, and various other drugs.^[12]^ Meanwhile, patients with other NSCLC subtypes may require different therapies based on their specific genetic profiles. Recognizing and accurately identifying ALK-positive NSCLC is crucial for delivering optimal, personalized treatment strategies to improve patient outcomes.

Alectinib has become the mainstay of treatment in ALK-positive NSCLC due to its long-term efficacy, high selectivity, and CNS-active ALK-inhibiting activity.^[11,13-15]^ Several clinical trials have demonstrated its efficacy against various ALK variants. However, a systematic review/meta-analysis is needed to compare these findings with similar drugs, such as brgatinib or crizotinib.^[11]^ Although alectinib has demonstrated long-term efficacy and safety, there are conflicting results from various studies, emphasizing, the need for a systematic review to showcase the results and provide a comprehensive review.^[16]^

The presence of conflicting studies regarding the efficacy and safety of alectinib and other ALK inhibitors represents a significant limitation in the current understanding of treatment options for ALK-positive NSCLC. To address this gap and provide clarity, we are conducting a comprehensive systematic review. This review aims to synthesize the available evidence, evaluate the discrepancies in findings, and ultimately enhance treatment decision-making and patient outcomes in this population.

## 2. Methods

Following the Preferred Reporting Items for Systematic Reviews and Meta-Analyses guidelines,^[17]^ a simple search strategy was employed to identify relevant PubMed, Medline, and PubMed Central studies. This strategy utilized a combination of medical subject headings and keywords relevant to the research question. The search was done under the parameters of including only the following clinical trials since inception and inclusion of only humans. The medical subject headings strategy comprised of “alectinib” (supplementary concept) and the initial search for the keyword: “NSCLC” identifies 5755 studies, “ALK” identifies 720 studies, and “alectinib” identifies 68 papers. Combining these terms with the Boolean operator “AND” yielded 47 potential studies for further review. A total of 6268 papers title and abstract screening. While checking for duplications, 52 articles were removed from the pooled articles. Screening of the article by title led to the selection of 47 articles, and further screening was done by reading the abstract and rigorous matching of the inclusion criteria; we identified 9 relevant studies for this analysis, which resulted in a total of 9 studies, including 9 clinical trials.

### 2.1. Inclusion criteria

- Randomized controlled trials (RCTs) and prospective observational studies.
- Articles comparing alectinib to other treatments in adult patients with ALK + NSCLC.
- ≥18 years patients with NSCLC with an identified ALK mutation.
- Articles evaluating alectinib as a treatment option for ALK + NSCLC.
- Articles with at least one of the following primary outcomes: OS, progression-free survival (PFS), objective response rate (ORR), or quality of life.
- Articles published in peer-reviewed English language journals.

### 2.2. Exclusion criteria

- Articles other than RCTs or prospective observational studies (e.g., technical analysis studies, expert opinions, case reports, case series, letters to the editors, retrospective studies).
- Articles include patients with other types of lung cancer or without confirmed ALK mutation.
- Articles do not report relevant outcomes.
- Articles published in other languages.
- Conference abstracts or gray literature.

Inclusion criteria for our systematic review will encompass RCTs and prospective observational studies that compare alectinib to other treatments in adult patients diagnosed with ALK-positive NSCLC. Eligible studies must focus on patients who are 18 years or older, have confirmed ALK mutations, and evaluate alectinib as a treatment option for this specific population. Furthermore, included articles should report at least one of the following primary outcomes: OS, PFS, ORR, or quality of life, and must be published in peer-reviewed English language journals. Conversely, we will exclude articles that do not meet these criteria, specifically those that are not RCTs or prospective observational studies, such as technical analysis studies, expert opinions, case reports, case series, letters to the editor, and retrospective studies. In addition, any articles that include patients with other types of lung cancer or without confirmed ALK mutations, do not report relevant outcomes, are published in languages other than English, or consist of conference abstracts or gray literature will also be excluded from our review.

### 2.3. Methodological rigor

This systematic review employed various strategies to minimize potential biases. During the study screening and selection phase, dual independent screening by 2 investigators helped reduce the risk of overlooking relevant studies or incorrectly including irrelevant ones. Any discrepancies were addressed by a third author, enhancing both consistency and accuracy.

In the data extraction phase, a similar independent review process helped mitigate errors, with a third reviewer verifying the extracted data for additional quality control. The quality assessment of the studies was conducted independently by 2 authors, and any disagreements were resolved through discussion, fostering a more objective evaluation. By focusing exclusively on high-quality clinical trials, the review aimed to limit the impact of methodological flaws. The quality appraisal of selected clinical trials was summarized according to established criteria (Table 1). Registration on PROSPERO UIN: CRD42024582275 provided transparency and accountability, which aided in mitigating publication bias and selective reporting.

**Table 1:** Summary of quality appraisal of selected clinical trials.

S.no.	Factor	Hotta et al^[16]^	Novello et al^[18]^	Peters et al^[19]^	Wu et al^[20]^	Yang et al^[21]^	Iwama et al^[15]^	Nishio et al^[22]^	Shaw et al^[14]^	Tamura et al^[11]^
1	Selection bias Random sequence generation	Low risk	Low risk	Low risk	Low risk	Low risk	NA	NA	NA	NA
2	Selection bias Allocation concealment	Low risk	Low risk	Low risk	Low risk	Low risk	NA	NA	NA	NA
3	Reporting bias Selective reporting	Low risk	Low risk	Low risk	Low risk	Low risk	Low risk	Low risk	Low risk	Low risk
4	Other bias Other sources of bias	Low risk	Low risk	Low risk	Low risk	Low risk	Low risk	Low risk	Low risk	Low risk
5	Performance bias Blinding (participants and personnel)	NA	NA	NA	NA	NA	NA	NA	NA	NA
6	Detection bias Blinding (outcome assessment)	NA	NA	NA	NA	NA	NA	NA	NA	NA
7	Attrition bias Incomplete outcome data	Low risk	Low risk	Low risk	Low risk	Low risk	Low risk	Low risk	Low risk	Low risk
	Total	Low risk	Low risk	Low risk	Low risk	Low risk	Low-Medium risk	Low-Medium risk	Low-Medium risk	Low-Medium risk

NA = not available.

While these methods significantly reduce the risk of biases, we acknowledge that no systematic review can be entirely free from them. Our paper is particularly susceptible to language bias, as we included only English studies, as well as the potential for unconscious researcher bias. However, we have made concerted efforts to minimize bias through transparent reporting of the search strategy and criteria, which enhances reproducibility and further supports the integrity of our review process.

### 2.4. Statistical analysis

Statistical analysis involves collecting, organizing, analyzing, and interpreting data to draw conclusions and inform decisions. It uses techniques such as descriptive statistics for summarization, inferential statistics for generalizations about a population, and hypothesis testing to evaluate claims. Systematic reviews does not involve mathematical analysis of data; rather, they focus on narrative synthesis of results of data.

## 3. Results

**Figure 2.**
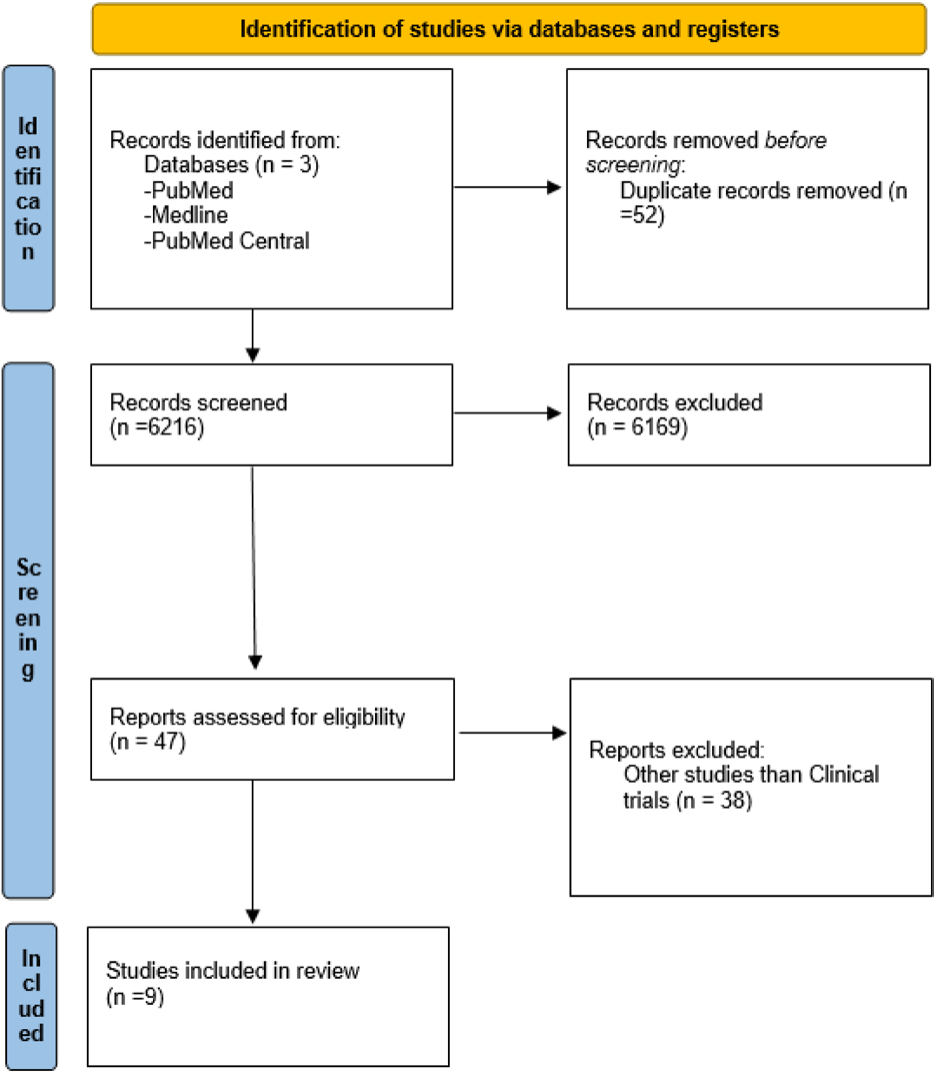
PRISMA flow diagram. PRISMA flow diagram, illustrating the process of study selection for a systematic review. The diagram summarizes the number of records identified, screened, assessed for eligibility, and ultimately shows the attrition of records at each stage and the final number of studies included in the review. PRISMA = Preferred Reporting Items for Systematic Reviews and Meta-Analyses.

Initial searches of PubMed, Medline, and PubMed Central identified 6268 papers. After removing duplicate articles (n = 52), 6216 articles thoroughly screened the titles for relevance to this study. Following this initial screening exclusion of 6169 articles, following a more comprehensive screening of the abstract, 47 articles met the criteria. These studies underwent a final screening based on full-text availability and relevance to this study. Ultimately, 9 studies were deemed suitable for this study and subjected to a standardized quality appraisal. The methodological rigor of this study was assessed using established criteria (Fig. 2).

**Figure 3.**
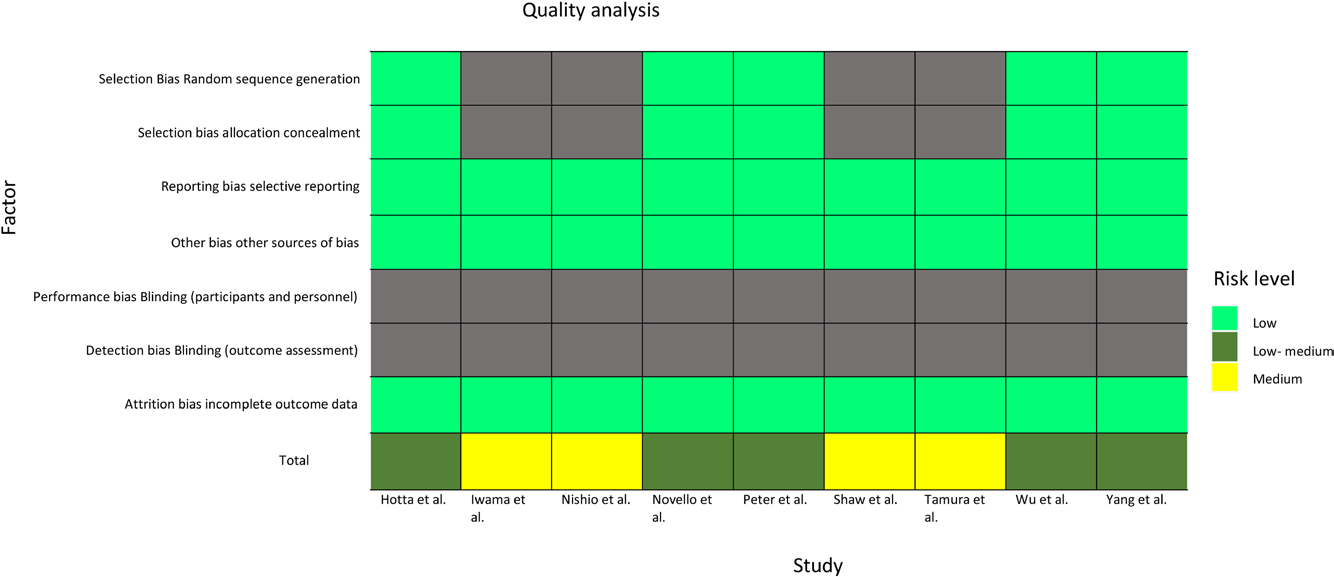
Quality appraisal. The risk of bias assessment for the various studies included in this systematic review. The assessment was conducted using the Cochrane risk of bias tool. Color coding for risk level indicators: Green showcases a low risk of bias. This indicates that the study is deemed to have a low risk of systematic error in that specific domain. Yellow showcases the risk of bias. This indicates that the study is deemed to have a medium risk of systematic error in that specific domain. Gray showcases risk of bias is not applicable. This signifies that the bias in that domain is not relevant to the specific study design. Study indicators: The columns represent individual studies included in the systemic review, while the rows correspond to the different domains that were assessed. Overall interpretation: A visual summary of the risk of bias across the studies and domains. A green indicator denotes low risk, a yellow indicator suggests medium risk, and a gray indicator reflects the risk of bias is not applicable.

The methodological rigor of the clinical trials in this analysis is displayed in a heatmap (Fig. 3).

Two independent investigators performed the quality appraisal of the study. To minimize bias, a third investigator was available to adjudicate any disagreements.

A total of 1403 patients with ALK-positive NSCLC were included in the analysis from 9 studies. A total of 836 patients were assigned to atelinib and 567 to other chemotherapeutic drugs.

The studies evaluated the 2 treatment modalities for short-term and long-term clinical outcomes, complications, valve durability, and patient health status.

## 4. Discussion

Considerable evidence suggests that alectinib holds significant promise as a therapeutic modality for ALK-positive patients with NSCLC. Despite the current dearth of comprehensive data on its overall efficacy and safety compared with alternative treatments, this knowledge gap presents an exciting opportunity for future research and potential breakthroughs. This discussion aims to compile the findings of the analyzed studies to clarify the potential of alectinib in the treatment of ALK-positive NSCLC.

The results of all the clinical trials used in the analysis of this systemic review are summarized (Table 2).

**Table 2:** Summary table.

S.no.	Papers	Year of publication	Purpose of research	Number of patients initially assigned	Result
1	Hotta et al^[16]^	July 14, 2022	To test if the overall survival improves with alectinib over crizotinib in ALK inhibitor naive Japanese patients with ALK-positive non-small-cell lung cancer	207 Total patients with alectinib (n = 103) and crizotinib (n = 104)	Results showed that alectinib did not increase the overall survival compared with crizotinib. However, alectinib remains the standard of care for ALK-positive non-small-cell lung cancer; this has been shown to delay the progression of metastasis mainly to the CNS regions
2	Wu et al^[20]^	April 10,2024	Compare the treatment of alectinib in ALK-positive resected non-small-cell lung cancer to the standard platinum-based chemotherapy	257 Total patients; alectinib (n = 130); chemotherapy(n = 127)	Based on previous studies, non-small-cell lung cancer is associated with increased brain metastases. However, treatment for ALK-positive non-small-cell lung cancer with alectinib is shown to decrease CNS metastases in comparison to the standard chemotherapy
3	Iwama et al^[15]^	February 23, 2017	Investigating the use of alectinib in ALK-rearranged Non-small-cell lung cancer and poor performance status	18 patients	The use of alectinib showed a performance improvement. It is also recommended in patients where chemotherapy is not recommended and in ALK-rearranged non-small-cell lung cancer
4	Shaw et al^[14]^	December 19, 2015	Understanding if alectinib can be used to treat ALK-positive crizotinib-resistant non-small-cell lung cancer	n = 87 patients	Prior studies have shown that chemotherapy and second generation ALK inhibitors help treat crizotinib-resistant non-small-cell lung cancer. However, chemotherapy has multiple side effects, including fatigue and myelosuppression. Alectinib is shown to have prolonged activity in comparison to certinib or brigatinib
5	Novello et al^[18]^	April 14, 2018	Testing to see if alectinib or chemotherapy is better for treating ALK-positive non-small-cell lung cancer	n = 107 patients; alectinib = 72; chemotherapy = 35	When comparing the progression-free survival, alectinib had a higher duration of 9.6 mo compared with the 1.4 mo with standard chemotherapy. CNS metastases are shown to be curbed with alectinib in comparison to chemotherapy as well
6	Yang et al^[21]^	August 12, 2023	Comparing the use of brigatinib versus alectinib in patients with disease progression using crizotinib	N = 248 patients; brigatinib = 125; alectinib = 123	Checking to see the overall survival rates, the 12-mo survival was 89% in brigatinib versus 96% in the alectinib group. The median time to intracranial response was also noted where the patient developed a CNS problem. The median time with brigatinib was 1.9 mo in comparison to the 3.5 mo with alectinib
7	Peters et al^[19]^	August 31, 2017	Understanding the use of alectinib versus crizotinib in untreated ALK-positive non-small-cell lung cancer	N = 303 patients; alectinib = 152; crizotinib = 151	The trial concluded that alectinib has a 53% lower risk of disease progression than crizotinib. The progression-free survival rate was 25.7 mo for alectinib compared with 10.4 mo for crizotinib
8	Nishio et al^[22]^	November 25, 2020	Testing to see if brigatinib helps treat Japanese patients who are ALK-positive for non-small-cell lung cancer who have been previously treated with alectinib or other TKI inhibitors	Main cohort (alectinib with or without crizotinib) = 47. All ALK-positive TKI refractory patients 72	Although brigatinib has multiple safety concerns, it has shown promising results in treating alectinib-refractory patients. However, this data was shown to be helpful in Japanese patients, and further trials with larger populations are recommended.
9	Tamura et al^[11]^	May 10, 2017	Understanding the effect of alectinib in patients with ALK-positive non-small-cell lung cancer over 3 years	N = 25 patients of the 46 patients in the trial on alectinib	Previously, in patients with ALK-positive non-small-cell lung cancer, treatment with chemotherapy or crizotinib was recommended. However, crizotinib showed an objective response rate of 74%, ceritinib with 63.7%, and alectinib with 93.5%. Based on this, alectinib is effective in treating these patients.

ALK = anaplastic lymphoma kinase, CNS = central nervous system, TKI = tyrosine kinase inhibitor.

### 4.1. Efficacy of alectinib

Iwama et al^[15]^ experimented with alectinib as the standard chemotherapy did not provide satisfactory results. The study concluded that the performance status had improved in these patients compared with the chemotherapy and recommended using alectinib in cases of failed unsatisfactory results.

Tamura et al^[11]^ *did a similar study* comparing crizotinib, ceritinib, and alectinib in patients with ALK-positive NSCLC, trying to ascertain their effectiveness and long-term impact (3 years); the study showed these drugs’ ORRs. The ORR for crizotinib, ceritinib, and alectinib are 74%, 63.7%, and 93.5%, respectively.

Interestingly, though, alectinib has significantly better ORRs when compared with crizotinib in terms of OS. Hotta et al^[16]^ showcased similar survival rates despite alectinib delaying the progression of CNS metastasis. This result of noncorrelating results of having a better ORR but similar OS could be due to the variations in genes, demographics, food, and environment. The study by Hotta et al^[16]^ was on ALK-naive Japanese patients who had tested ALK-positive for NSCLC.

In the Japanese demographic, Nishio et al's^[22]^ studies observed that alectinib generated favorable outcomes despite the development of resistance, prompting the need for alternative chemotherapeutic interventions. Brigatinib, although practical, poses safety concerns. However, alectinib’s positive outcomes in this specific population provide reassurance about its effectiveness in treating ALK-positive NSCLC.

Based on the findings of these 2 studies,^[16,22]^ alectinib is deemed essential in treating NSCLC, particularly in preventing CNS metastasis in ALK-positive Japanese patients, with a minimal risk of alectinib refractory development. Our strong recommendation is that future studies include a more diverse population to determine the generalizability of these findings and to identify any potential confounding effects on the efficacy of ALK-positive NSCLC.

Traditionally, platinum-based chemotherapeutic drugs were the standard of care for NSCLC. Recent discoveries of newer drugs, such as the ALK tyrosine kinase inhibitors, led to a study comparing platinum-based chemotherapy’s efficacy against the novel alectinib in patients with ALK-positive NSCLC.

Wu et al^[20]^ and Novello et al^[18]^ showcased that ALK-positive resected NSCLC alectinib had significantly decreased rates in CNS metastases treated with platinum-based chemotherapy or alectinib. The PFS in patients taking alectinib and chemotherapy, as showcased by Novello et al,^[18]^ showed a higher duration of 9.6 months compared with 1.4 months in chemotherapy. This evidence should help physicians and future researchers consider using alectinib as a more standard treatment than chemotherapy in patients with ALK-positive NSCLC.

Crizotinib is a hepatocyte growth factor receptor tyrosine kinase inhibitor; crizotinib has a strong association with developing resistance, and a study conducting research recommends that alectinib has showcased promising results compared with other ALK tyrosine kinase inhibitors, with long duration but also expressing multiple side effects, such as myelosuppression.^[14]^ Another similar study compared the effectiveness of alectinib versus brigatinib in patients with disease progression who had been using crizotinib showcased 2 critical factors: OS rates in 12 months and median time to intracranial response. First, the OS rates of alectinib were 96% compared with brigatinib, which was around 89%. Second, the median time to intracranial response for alectinib was around 3.5 months in comparison to 1.9 months for brigatinib; this showed delayed progression of CNS metastasis and increased survival in patients with crizotinib resistance when taking alectinib rather than begatinib.^[21]^

Based on the evidence, alectinib has demonstrated remarkable efficacy, establishing itself as a crucial drug in treating ALK-positive NSCLC, surpassing other treatment modalities in its performance.

Efficacy variations of alectinib has been observed across different age groups in treating ALK-positive NSCLC, particularly highlighting the challenges faced by elderly patients. Older individuals often present with age-related comorbidities that may hinder their performance status and overall treatment outcomes.^[15]^ However, another study has showcased improvements in investigator-assessed PFS for alectinib showcasing effectiveness even in older populations.^[18]^ Overall, understanding these efficacy variations is critical for optimizing treatment strategies and improving patient outcomes across different age demographics.

Alectinib has demonstrated significant effectiveness as an adjuvant therapy in completely resected stage IB, II, and IIIA ALK-positive NSCLC, showing a marked improvement in disease-free survival when compared with chemotherapy options. This enhancement in outcomes is particularly crucial in the early stages of the disease.^[20]^ In addition, alectinib has also proven to be highly efficacious in advanced or metastatic ALK-positive NSCLC, specifically in stages IIIB and IV, where it has shown promising results in controlling disease progression and improving overall patient prognosis. These findings underscore the importance of alectinib in both early and advanced stages of ALK-positive NSCLC, making it a vital component of targeted therapy in this patient population.

Alectinib has demonstrated significant efficacy in patients who have previously progressed on crizotinib, as highlighted in the ALUR study, which showed superior PFS compared with chemotherapy for those with ALK-positive NSCLC.^18]^ Specifically, the median duration of response for alectinib was reported at 13.5 months for crizotinib-failed patients, outperforming other next-generation ALK inhibitors like ceritinib and brigatinib, which had a median duration of responses of 8.2 and 9.3 months, respectively. Alectinib’s advantages are also indicated in terms of tolerability, as suggested by single-arm trials.^[14]^ However, when assessing efficacy in alectinib-refractory populations, brigatinib has displayed meaningful activity, particularly against brain metastases, capitalizing on its broad-spectrum efficacy against various ALK mutations linked to resistance.^[17]^

While alectinib showed superior PFS, OS did not significantly differ between treatments, potentially due to a considerable crossover of patients from crizotinib to alectinib after disease progression. This suggests that prior treatments, including chemotherapy, can significantly shape the efficacy of subsequent ALK inhibitors. ALTA-3 indicated that patients with lower rates of prior chemotherapy experienced better outcomes, further emphasizing how treatment history influences efficacy in ALK-targeted therapies.^[21]^

### 4.2. Safety

Concerning safety, alectinib is a well-tolerated medication compared with its counterparts, and it is easily manageable compared with the traditional standard of care of chemotherapy.^[22,20]^

Alectinib, while effective for treating ALK-positive NSCLC, is associated with several common side effects that clinicians should be mindful of when managing patient care.

Some of the most common side effects of alectinib in patients with NSCLC are as follows. Constipation is one of the most frequently reported adverse events, typically presenting as mild to moderate in severity (grade 1–2).^[14,19,22]^ Fatigue is another commonly noted side effect, also generally of mild to moderate severity.^[14,22]^ In addition, a significant proportion of patients experience muscle aches and pains (myalgia).^[14,19,22]^

Peripheral edema is another concern, alongside elevated levels of creatine phosphokinase, which can indicate muscle damage;^[14,22,20]^ nearly 30% of patients exhibit grade 3 or greater elevations in creatine phosphokinase levels.^[19]^ Furthermore, increases in liver enzymes, such as aspartate aminotransferase and alanine aminotransferase, have been documented,^[11,14,21,22]^ with grade 3 elevations observed in ≈5% to 6% of patients.^[14]^ Patients may also report alterations in taste perception (dysgeusia), which can affect dietary intake and overall well-being.^[11,22,20]^

When using alectinib, clinicians should be aware of several rare side effects that may impact patient management. Some of the potentially severe adverse effects of alectinib is interstitial lung disease, which has not been observed in clinical trials with alectinib as of yet, but it is noteworthy that this condition has a 2% occurrence rate with brigatinib, highlighting the potential for similar risks in alectinib.^[19]^ In addition, hemorrhage represents another potential complication, particularly in patients undergoing anticoagulant therapy, and is believed to be associated with the use of alectinib.^[14]^ Furthermore, a decrease in neutrophil counts has been noted as a rare side effect, necessitating vigilance in monitoring blood parameters in patients receiving this treatment.^[14,22]^ Awareness of these rare side effects can help clinicians provide comprehensive care and prompt intervention when necessary.

Most adverse events can be effectively managed through dose adjustments or supportive care measures.^[14,18,22,20]^ The low incidence of severe adverse events and the effectiveness of management strategies are reflected in the low rates of treatment discontinuation due to adverse events in all clinical trials.

### 4.3. Limitations and future considerations

It is important to note that the studies assessing safety and efficacy are of a considerably small size. Substantial clinical trials with diversified patients might be required to generalize our concerns about using alectinib in ALK-positive NSCLC. Furthermore, more research is needed to improve the current understanding of factors associated with alectinib becoming refractory or to determine if alectinib innately becomes refractory after a period of use. Even the evidence comparing alectinib to brigatinib is limited. Further research in this area would lead to a better understanding of treating ALK-positive NSCLC. There is also a need for further studies, in addition to biopsy studies and scans, both before and after treatment with alectinib. These studies help quantify how much superior/noninferior alectinib performs compared with other treatment options.

## 5. Conclusion

This comprehensive systematic review meticulously evaluated alectinib and compared its efficiency and safety with the other plausible treatment modalities. By critically analyzing the current evidence, we conclude that alectinib is a promising treatment option in patients with ALK-positive NSCLC. The results indicate that alectinib is a highly effective treatment option, with superior response rates, prolonged PFS, and improved control of CNS metastases compared with alternative therapies. While alectinib is generally well-tolerated, further extensive studies are necessary to comprehensively evaluate its long-term safety profile. Compared with other treatment options, alectinib has proved to be a plausible option in patients with a wide range of clinical scenarios and demographics. In order to optimize patient-centered health care, future research will focus on early positive biomarkers, combination therapies, and cost-effectiveness assessments.

## Acknowledgments

The authors would like to acknowledge the use of Grammarly and Paperpal to improve the grammatical accuracy and clarity of this manuscript and no other external support was received for this study.

## Author contributions

**Conceptualization:** Monica Kharat, Harshitha Nookala, Jabez David John, Tirath Patel.

**Methodology:** Monica Kharat, Harshitha Nookala, Jabez David John, Tirath Patel.

**Project administration:** Monica Kharat, Jabez David John, Tirath Patel, Nikhilesh Anand.

**Writing – original draft:** Monica Kharat, Harshitha Nookala, Jabez David John, Tirath Patel, Sonya Emmanuel.

**Formal analysis:** Harshitha Nookala, Jabez David John, Tirath Patel, Pragnesh Kalyandrug.

**Data curation:** Pragnesh Kalyandrug, Sonya Emmanuel, Anaya Noor, Dhruv Jignesh Kumar Halani.

**Visualization:** Pragnesh Kalyandrug, Sonya Emmanuel, Anaya Noor, Dhruv Jignesh Kumar Halani.

**Writing – review & editing:** Abhishek Goyal, Nikhilesh Anand, Bharathi Gadad, Richard M. Millis.

**Supervision:** Nikhilesh Anand, Bharathi Gadad, Richard M. Millis.
